# *BornAgain*: software for simulating and fitting grazing-incidence small-angle scattering

**DOI:** 10.1107/S1600576719016789

**Published:** 2020-02-01

**Authors:** Gennady Pospelov, Walter Van Herck, Jan Burle, Juan M. Carmona Loaiza, Céline Durniak, Jonathan M. Fisher, Marina Ganeva, Dmitry Yurov, Joachim Wuttke

**Affiliations:** a Jülich Centre for Neutron Science (JCNS) at Heinz Maier-Leibnitz Zentrum (MLZ), Forschungszentrum Jülich GmbH, Lichtenbergstrasse 1, Garching, 85748, Germany

**Keywords:** grazing-incidence small-angle scattering (GISAS), X-ray scattering, neutron scattering, simulation, software

## Abstract

*BornAgain* is a free and open-source multi-platform software framework for simulating and fitting X-ray and neutron reflectometry, off-specular scattering, and grazing-incidence small-angle scattering (GISAS). This paper concentrates on GISAS. Support for reflectometry and off-specular scattering has been added more recently, is still under intense development and will be described in a later publication. *BornAgain* supports neutron polarization and magnetic scattering. Users can define sample and instrument models through Python scripting. A large subset of the functionality is also available through a graphical user interface. This paper describes the software in terms of the realized non-functional and functional requirements. The web site https://www.bornagainproject.org/ provides further documentation.

## Abstract 2

*BornAgain* is a free and open-source multi-platform software framework for simulating and fitting X-ray and neutron reflectometry, off-specular scattering, and grazing-incidence small-angle scattering (GISAS). This paper concentrates on GISAS.

## 1. Introduction   

X-ray and neutron reflectometry, off-specular scattering, and grazing-incidence small-angle scattering (GISAS) are closely related experimental techniques for the structural characterization of thin films and interfaces. They yield feature-rich data that depend in complicated ways on the scattering-length distribution in the sample. Computing the latter from the former is an inverse problem that is hard for reflectometry and underdetermined for scattering.

In favorable cases, it is possible to index diffraction peaks, or to interpret other salient features, and to directly extract some parameters from the data, or fit some mathematical approximation *ad hoc*. More often, however, data analysis is done by parametric modeling, computer simulation and fitting: one chooses a real-space model which is compatible with all that is known about the sample structure. Then one simulates the experiment by computing the reflected or scattered intensity. If there is qualitative resemblance with the measured data one can further improve the agreement by manually or automatically optimizing some model parameters. Obviously, this workflow needs to be supported by adequate software. Besides data fitting, such software can also be used for training and for experiment planning.

In this paper, we present the software *BornAgain*, which we have been developing since 2012. The *BornAgain* project was initiated with the aim of superseding unmaintained legacy software (Section 2) and of satisfying requests for additional functionality. Its name alludes to the distorted-wave Born approximation (DWBA), which is indispensable for describing scattering under grazing incidence. *BornAgain* is intended for experimentalists who come from different disciplines, work in different application domains, and have quite different degrees of knowledge, experience and interest in scattering methodology, data analysis and computing.

*BornAgain* is the first, and to date the largest, project of the Scientific Computing Group of Heinz Maier-Leibnitz Zentrum (MLZ) Garching. MLZ is committed to ensuring continued maintenance. In the data analysis workpackage of SINE2020 (https://www.sine2020.eu/), European neutron scattering centers have agreed on a division of tasks. In recognition of its initial investment in *BornAgain*, MLZ was made responsible for the entire field of reflectometry, including off-specular scattering and GISAS. In fulfillment thereof, we are currently extending *BornAgain* to specular reflectometry and off-specular scattering. This will be described in a sequel paper (see Section 8.1). Here, we concentrate on GISAS.

The most comprehensive review of this method, as far as X-rays are concerned (GISAXS), remains that of Renaud *et al.* (2009 ▸). For neutron scattering (GISANS) in soft matter, see the work of Müller-Buschbaum (2013 ▸); for polarized GISANS in magnetism research, see the theoretical framework by Kentzinger *et al.* (2008 ▸) and the reviews by Paul (2012 ▸) or Toperverg (2015 ▸). For an update that treats X-rays and neutrons equally, see the work of Hexemer & Müller-Buschbaum (2015 ▸). The most recent review is by Jaksch *et al.* (2019 ▸). For feature extraction and real-time analysis, a good example is provided by the work of Schwartzkopf *et al.* (2013 ▸, 2015 ▸).

The present paper gives a broad overview of the *BornAgain* project as per release 1.16 of August 2019. It is not intended as a user manual and does not repeat details from the online documentation (Section 3.7). It focuses on the general concepts and on the software engineering aspects of the project.

After reviewing extant software (Section 2), we present *BornAgain* along with an analysis of requirements. This implies by no means (Parnas & Clements, 1986 ▸) that we worked linearly from requirements to implementation. Rather, the development of *BornAgain* is an iterative process (Meyer, 2014 ▸) that critically depends on our learning from user feedback. Non-functional requirements (*e.g.* Glinz, 2007 ▸), and choices made in response to them, are discussed in Section 3. Sections 4 to 6 are concerned with functional requirements: Section 4 introduces the graphical and scripting user interfaces, Section 5 gives an overview of the physics implemented in *BornAgain*, and Section 6 discusses data processing and fitting. Finally, Section 7 presents examples of *BornAgain* used in published experimental work, and Section 8 gives a brief outlook on future extensions of *BornAgain*.

## 2. Extant software   

To work out functional requirements for *BornAgain*, we took advantage of the thoughts and experiences embodied in preceding software projects. A full list of reflectometry and GISAS software can be found in the GISAXS wiki (http://gisaxs.com/index.php/Main_Page). In the following we discuss projects that influenced the design or functionality of *BornAgain*, or that are otherwise important.

### 2.1. *IsGISAXS*   

The Fortran program *IsGISAXS* (Lazzari, 2002 ▸, 2006 ▸) was the first widely used software in the field. Its lasting importance for the analysis of GISAS data is attested by the many hundreds of citations. The source code is available for non-commercial use.

*IsGISAXS* supports substrate–air and substrate–overlayer–air models with embedded particles (islands, inclusions or holes) of various geometrical shapes. The embedded particles either form a paracrystal or are disordered as specified by an interference function or by real-space coordinates. The latest release, 2.6 of 2006, brought support for a graded interface. For fitting, the Levenberg–Marquardt algorithm and simulated annealing are implemented. Simulation and fit are parameterized through fixed-format files.

To facilitate migration towards *BornAgain*, we re-implemented almost the entire functionality of *IsGISAXS*. This work was completed with *BornAgain* 1.8, which brought support for graded layers. All re-implemented functionality has been verified by comparing simulation results from both codes.

### 2.2. *NANOCELL*   

*NANOCELL* (Tate *et al.*, 2006 ▸), written in the proprietary *Mathematica* language, addressed small-angle X-ray scattering and GISAXS by self-assembled crystalline nanomaterials. The code is available on request.

### 2.3. *FitGISAXS*   

*FitGISAXS* (Babonneau, 2010 ▸), written in the proprietary language *IGOR Pro*, provides DWBA computation for an arbitrary number of layers. The complex amplitudes of downwards and upwards traveling waves are computed using a matrix formalism (Abelès, 1950 ▸). For fitting, Levenberg–Marquardt and orthogonal distance regression are supported, and there is a simple graphical user interface (GUI).

### 2.4. *HipGISAXS*   

The project *HipGISAXS* (Chourou *et al.*, 2013 ▸; Sarje *et al.*, 2016 ▸) mainly addressed form-factor computation for particles of arbitrary shape, using fine surface triangulation. Emphasis is on massively parallel execution on CPUs and GPUs, using MPI, CUDA and OpenMP. The C++ code is available under a non-commercial license.

## 3. Non-functional requirements and choices   

Many non-functional requirements are very similar for all kinds of scientific software. Therefore, wherever possible, we follow the emergent consensus of the research software community (Prlić & Procter, 2012 ▸; Wilson *et al.*, 2014 ▸; Jiménez *et al.*, 2017 ▸). In turn, many of our choices have found their way into the recently compiled ‘Standards and Guidelines’ for neutron scattering data analysis software (Markvardsen, 2017 ▸).

### 3.1. License   

*BornAgain* is released as free and open source under the GNU General Public License, version 3 or higher. Open code is a necessary condition for verifiable research (Peng, 2011 ▸; Ince *et al.*, 2012 ▸; Joppa *et al.*, 2013 ▸; Hinsen, 2016 ▸). Furthermore, free and open code enables advanced users to adapt it to their own needs (Gentleman *et al.*, 2004 ▸), encourages collaboration (Willinsky, 2005 ▸) and ensures long-term sustainability by authorizing forking, which is needed as a last resort when collaboration fails or maintainers become unresponsive (Nyman & Lindman, 2013 ▸).

### 3.2. Version control and collaborative workflow   

All source code is under version control, using *git*. This includes all build and test scripts, and also the LaTeX sources of the physics manual. The repository is currently hosted at https://github.com/scgmlz/BornAgain.

Our collaborative workflow combines the branching model of Driessen (2010 ▸) with the pull request machinery of GitHub. To work on one specific issue (*e.g.* correct a bug, improve performance, implement new functionality or refactor the code in preparation for further modifications), a developer forks the shared develop branch, spawns a feature branch, modifies the code, runs all tests and finally opens a pull request. Another developer will then review the modifications, possibly demand corrections and finally merge the feature branch into the shared develop branch. External contributors need only to register with GitHub; then they can develop and submit pull requests in exactly the same way as the *BornAgain* core team. Near the end of a release cycle, the develop branch is frozen, intensely tested and debugged as necessary. Then a release branch is spawned, finally merged into the master branch and tagged with the new version number, which follows the semantic versioning scheme (Preston-Werner, https://semver.org/). When necessary, bugs are corrected in hotfix releases.

### 3.3. Programming languages   

*BornAgain* is written in C++. This choice was motivated by the need for optimum computational performance, by the wish to implement Core and GUI in one and the same language, and by the fact that no other suitable language is sufficiently well known in our community. In the course of the project, we migrated to the much improved idiom of C++11, then to C++14. Our nightly tests ensure that *BornAgain* passes under three different compilers, *gcc*, *clang* and *Visual Studio*.

**Figure 1:**
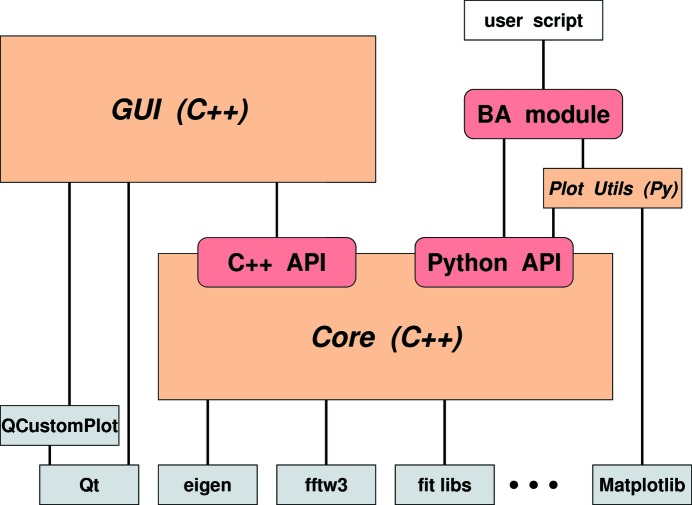
Overall code architecture (red: interfaces; orange: *BornAgain* components; gray: external dependencies; white: user code). The *BornAgain* Core can be controlled either from a GUI or from Python3 scripts. ‘BA module’ stands for the *BornAgain* Python module, which comprises a thin Python layer with plot utilities and the automatically generated Core wrapper.

The *BornAgain* Python module (Section 4.2) is made from wrapper code that is distributed as part of the *BornAgain* source, but can be regenerated at any moment by running the open-source tool *Swig*. Besides this wrapper code, the *BornAgain* Python module also comprises some utility functions, written in Python, that facilitate and standardize use of the standard Python plotting library *Matplotlib*. The resulting code architecture is outlined in Fig. 1 ▸. The Core comprises about 60k lines of code, the GUI 90k.

### 3.4. Parallel computation   

To simulate a GISAS detector image, *BornAgain* runs a loop over all pixels. For each pixel, it computes the scattering cross section 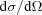 (Section 5.2) at a final wavevector 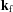 given by the pixel’s coordinate. This algorithm lends itself to easy parallelization, since the computations at different 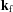 are completely independent of each other.

*BornAgain* fully supports multi-threading. By default, all processor cores of the CPU are used. *BornAgain* can also be compiled with *OpenMPI* for multi-node computation on high-performance clusters.

### 3.5. Cross-platform support and build system   

*BornAgain* is actively supported for the three operating systems Linux, MacOS and Microsoft Windows, the last only in 64 bit versions. The download page provides installers for Mac and Windows. Installers for Linux may be added later; for the time being, Linux users have to compile from source.

Sources are distributed as a tarball. They come with input scripts for the meta-build system *CMake*. *CMake* generates makefiles for the Unix *Make* program, generator files for the *Ninja* build system, or project files for integrated development environments (IDEs), among them Microsoft *Visual Studio*. Thereby *CMake* generalizes the configure step of Unix-only source distributions, and enables native compilation under Microsoft Windows.

*CMake* searches for external library dependencies. These are currently the GNU scientific library for mathematical routines, including some fit algorithms, *fftw3* for fast Fourier transform (Frigo & Johnson, 2005 ▸), *libtiff* for reading some detector images, some components of *Boost*, mostly for input/output processing, and *Qt5* for the GUI. Some other libraries are distributed along with the *BornAgain* sources in a ThirdParty directory, mainly because they are not easily available in well-configured packages for all three supported platforms. These include *Google tests*, *Minuit2* (a C++ rewrite of the Fortran fit library *MINUIT*, maintained by CERN and distributed within CERN’s *ROOT* library, but also available in a standalone version) and *QCustomPlot*. The library *Eigen* for vector and matrix algebra is included as a *git* submodule.

### 3.6. Tests   

The *BornAgain* sources contain different kinds of tests. They are all built and run using the *CMake/CTest* system.

Nearly 5000 EXPECT clauses in over 700 unit tests check specific functionality of over 150 classes. The unit test machinery relies on the *Googletest* C++ testing and mocking framework. GUI-related tests additionally depend on the introspection machinery of *QtTest*.

The functional tests, except a few special ones, are all regression tests. They run a full simulation on a specific instrument and sample model, and compare the result with a reference image that is also part of the *BornAgain* sources. These tests ensure that modifications of the code do not compromise the correctness and accuracy of simulation outcomes. They link back the current code to the earliest, still very simple versions of *BornAgain*, and to *IsGISAXS*. For speed reasons, the simulated detector has only 25 × 25 pixels.

The models and reference data of the Core regression tests are also used in Python and GUI functional tests. In the Python tests, C++ models are exported to Python, then a simulation is run under Python, which implicitly translates the model back to C++, and the final outcome is compared with the reference image. Similarly, in the GUI tests, C++ models are extended into GUI models, and simulations are run from the Born-AgainGUI library, without launching an actual GUI. In these ways, about 70 test cases can be run from C++, from Python and through the GUI machinery.

### 3.7. Online services   

The project web site https://www.bornagainproject.org/ provides detailed information on how to download, install and run *BornAgain*. It offers extensive tutorials, especially on how to set up various sample models. To produce these web pages, we use the static web site generator *Hugo*. All page sources are under version control. All code examples in the tutorials are also included in the *BornAgain* source distribution and are covered by tests, so that at any time all tests are guaranteed to work with the latest software release.

The web site provides links to the download location, to the *git* repository, to the issue tracker and to the subscription form of an announcements mailing list. For direct inquiries to the maintainers, a contact mail address is given. In the future, the API (application programming interface) reference, automatically generated by *Doxygen*, will be tightly integrated with usage examples and documentation of the implemented physics.

### 3.8. Outreach   

To meet user needs, we depend on feedback. The *Born­Again* team is in close contact with reflectometry scientists at our neutron source FRM II. To reach the GISAS community at large, we presented *BornAgain* in talks, posters and tutorials at numerous conferences in Europe, North America and East Asia. In 2013 we invited experienced users and developers of extant software to a workshop on GISAS data analysis.

In 2016 and 2018, we organized the first and second *BornAgain* School and User Meeting. Each time, more than 30 experimentalists attended. They were given an in-depth introduction to *BornAgain*, complemented by practical exercises. Advanced users presented their results. We intend to continue these meetings every two years.

## 4. User interfaces   

### 4.1. GUI   

*BornAgain* comes with a GUI. Based on the library *Qt5*, it has a native look and feel under all three supported operating systems (Section 3.5). It allows users to view experimental and simulated data, to set up and parameterize physical models, and to run fits.

**Figure 2:**
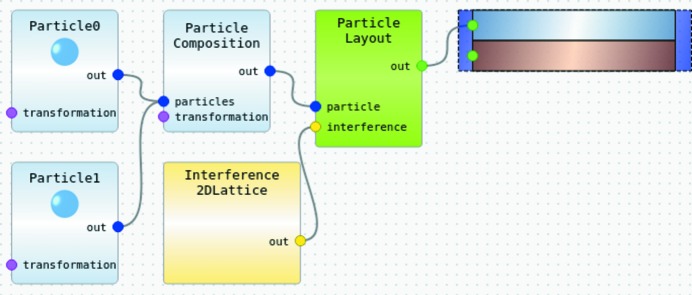
Representation of a sample in the GUI model editor. The hierarchical sample construction starts from the element in the upper-right corner, which just represents air above a substrate. The semi-infinite air layer is decorated with a particle layout, which combines a particle composition motif, composed of two spherical particles, with an inter-motif correlation function, namely a 2D lattice.

A graphical sample editor, vaguely inspired by the *LabVIEW* way of programming, allows users to assemble multilayer models by drag and drop. Model components like embedded particles or inter-particle correlation functions are represented by boxes that are connected by flexible lines to their parent components, as shown in Fig. 2 ▸.

**Figure 3:**
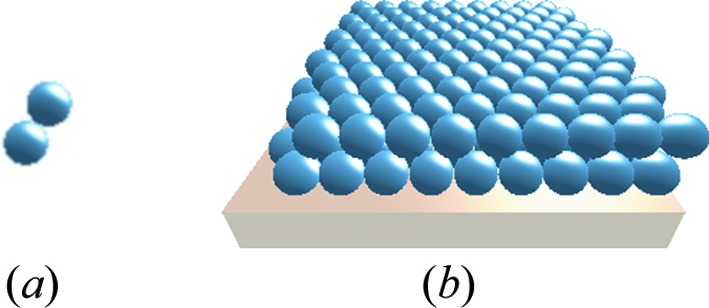
Real-space representation of the sample model of Fig. 2 ▸, a hexagonal bilayer on top of a substrate: (*a*) particle composition motif, (*b*) entire sample. In the GUI, these 3D views can be interactively rotated. The resulting GISAS pattern is shown in Fig. 11.

Simultaneously, the real-space structure of the sample, or any of its components, can be shown in an interactive 3D visualization (Fig. 3 ▸). This provides users with feedback as to whether the model, constructed in abstract terms in the sample editor, corresponds to their actual intentions. Moreover, a real-space representation can be helpful when communicating scientific results to audiences that are not familiar with the reciprocal-space thinking of scattering practitioners.

All model parameters can also be seen and set from a tree view. To explore their impact upon the simulated GISAS pattern, it is possible to modify parameter values with sliders. Unless the model is excessively computing intensive, the outcome view is kept up to date without noticeable delay.

All model and parameter choices made in an interactive GUI session can be stored in XML and can be reloaded in another GUI session. They can also be stored as a Python script, which can then be run from the command line, independently of the GUI. This helps users who reach the limits of GUI functionality to get started with using *BornAgain* from Python, since it is much easier to edit a given script than to write one from scratch.

### 4.2. Python interface   

The Python API enables users to run simulations and fits from the high-level programming language Python3. This can be done either in interactive sessions (possibly in a *Jupyter* notebook) or by executing a script. Once Python and *BornAgain* are installed on a computer, the simple command import bornagain as ba in a Python session or script is sufficient to make all the functionality of *BornAgain* available through function calls with the prefix ba.

The online documentation comprises numerous example scripts. For each script, the implemented model is explained and the obtained simulation result is shown. The same scripts are also contained in the *BornAgain* source tree. Typically, users will start from some example script and then gradually adapt it to their own needs. Another way to get started with scripting was previously mentioned in Section 4.1 – namely, build a model in the GUI and export it as a Python script.

Scripting is more versatile than the GUI and provides functionality that is not yet available in the GUI, or never will be. For instance, one can set up arbitrarily complicated combined and constrained fits. One can program the batch processing of huge data sets. One can react to a simulation or fit outcome through control clauses.

One can also extend the functionality of the *BornAgain* Core, for instance by adding particle form factors or correlation functions. Implementing such functionality in Python is less computing efficient than in C++, but in terms of development effort it can be an efficient way of rapid prototyping.

## 5. Simulated physics   

In this section we summarize the physical models implemented in *BornAgain*. After discussing geometric conventions, instrument models and detector coordinates (Section 5.1), we review the DWBA scattering cross section (Section 5.2) and add a word of caution about simulated peaks that are narrower than the detector bins (Section 5.3). We then discuss beam propagation in multilayers (Section 5.4) and the parameterization of refractive indices (Section 5.5). Finally, we present implemented models for sample inhomogeneities (Sections 5.6–5.13).

### 5.1. Geometrical conventions and detector models   

**Figure 4:**
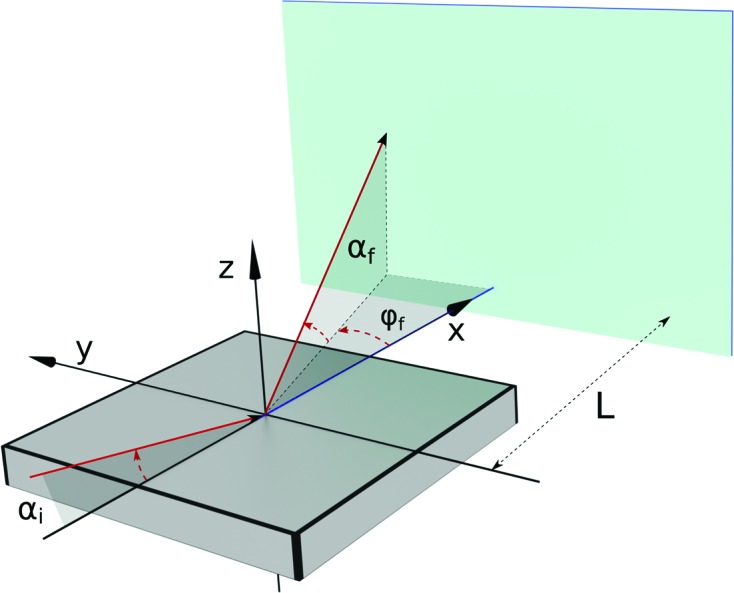
Geometric conventions in *BornAgain*. The average sample surface is the *xy* plane. The mean incident beam lies in the *xz* plane. The detector, at distance *L* from the origin, is perpendicular to 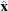.

To describe GISAS geometry, we follow well-established conventions (Fig. 4 ▸). ‘Grazing incidence’ is understood relative to an average sample surface, always identified with the *xy* plane and called ‘horizontal’, no matter how it is oriented in laboratory space. The mean incident beam lies in the *xz* plane and originates from the quadrant *x* < 0, *z* > 0. The grazing angle is α; the azimuth angle φ is the deflection away from the *xz* plane.

Currently, instrument imperfections are modeled in reciprocal space only. Real-space characteristics like beam profile and finite sample surface will be added in future extensions for reflectometry (Section 8.1; compare Adlmann *et al.*, 2018 ▸).

By default, *BornAgain* assumes a perfectly monochromatic and collimated incoming beam. This can be overwritten by explicit choices for the distributions of incoming wavelength, inclination and azimuth angle. So we admit 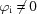, but the mean azimuth is 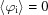.

Two detector geometries are supported: spherical and flat. A spherical detector is fairly unrealistic, but provides a means to visualize scattering patterns as functions of orthogonal coordinates 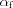 and 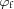. The flat detector should be used when simulations are to be compared with experimental data from actual area detectors (*e.g.* Lehmann *et al.*, 2011 ▸; Ponchut *et al.*, 2011 ▸). By default, the detector lies in the *yz* plane, but can be tilted as specified by its normal vector **n**. In this way, we also support grazing-incidence wide-angle scattering.

**Figure 5:**
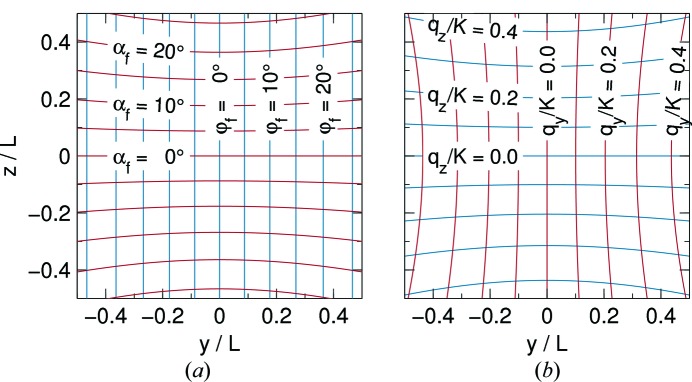
Scattering coordinate systems. Detector coordinates *y*, *z* are shown relative to the detector distance *L*. The red and blue curves are lines of constant (*a*) scattered beam angles 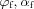, and (*b*) scattering vector components 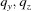, the latter relative to the radiation wavenumber 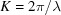.

The natural pixel coordinates of the flat detector are *y*, *z* in units of mm. To facilitate physical interpretation, intensity maps can also be labeled with 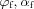 or 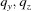, which are approximated proportional to *y*, *z*; no re-binning is done. As Fig. 5 ▸ illustrates, this is adequate only in the small-angle limit. Note also that 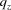 is defined with respect to the direct (transmitted) beam, while the reflected terms in the GISAS cross section imply that some of the scattering actually occurs at 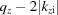.

A finite detector resolution can be activated by choosing a Gaussian blur. The instrument model also offers a choice to add constant or Poisson noise to simulated detector images.

### 5.2. DWBA cross section   

The elastic scattering of neutrons and X-rays is governed by the wave equation
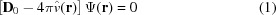
with the vacuum wave operator
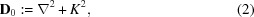
at wavenumber *K*, and the potential (or scattering-length density, SLD)
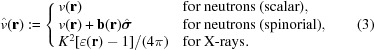
The amplitude Ψ represents the scalar or spinorial neutron wavefunction, or the electric field, as applicable. The caret notation 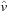 indicates, in the spinor case, a 2 × 2 matrix; the Pauli vector 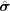 is composed of the three Pauli matrices 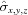. Fermi’s pseudopotential is used in the rescaled form:
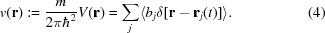
The sum runs over all nuclei; 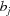 is the bound scattering length (Sears, 1992 ▸). The magnetic moment μ of the neutron couples to the magnetic induction **B** (Mezei, 1986 ▸; Majkrzak *et al.*, 2006 ▸), 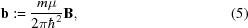
where *m* is the neutron mass and ℏ is the reduced Planck constant. For the X-ray case, the simple form (2) depends on a few standard assumptions: non-conducting medium, hence no currents and no free charges, and no fast fluctuations of the permittivity ∊(**r**), except for jumps at layer interfaces.

In the DWBA, applied to layered samples (Dietrich & Wagner, 1984 ▸, 1985 ▸), the potential is split as
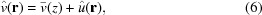
where 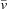 depends only on depth *z*, while 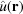 collects all fluctuations in *x* and *y*. The function 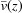 can also be expressed through the refractive index:
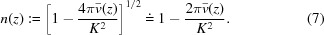
With the distorted-wave operator 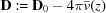, the wave equation (1) can be recast as
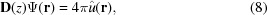
where the term on the right-hand side shall be treated as a small perturbation. In first order of the Rayleigh–Born expansion, the scattering cross section is
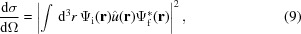
where 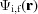 are solutions of the unperturbed, homogeneous wave equation
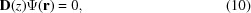
subject to the boundary condition that they are plane waves at distant source and detector locations, respectively.

### 5.3. Caution about narrow structure-factor peaks   

At this point, a word of caution is in order. In *BornAgain*, by default the DWBA cross section is only evaluated at the centers of the detector bins. If the sample model results in a structure factor that varies substantially within one bin, then large discretization errors must be expected.

*BornAgain* currently has no mechanism to warn about this situation. It is entirely the responsibility of users to anticipate the possibility of narrow peaks and to take appropriate precautions. Since narrow peaks in **q** arise from long-range correlations in real space, one ought to be suspicious about thick layers, large embedded particles and especially crystalline order.

We recommend three ways to deal with narrow peaks. (i) Check whether the simulation outcome is stable under an increased or reduced number of bins.^1^ (ii) Run *BornAgain* in Monte Carlo mode to average for each bin over several **q** chosen at random. (iii) Extend the model to account for finite crystal size or finite coherence length, as discussed in Section 5.12.

### 5.4. Distorted waves for multilayers   

The boundary condition for the incident wave Ψ_i_ is determined by its vacuum wavevector **k**_i_. The elastically scattered wave Ψ_f_ must be traced back from each single detector pixel; its vacuum wavevector **k**_f_ is given by the modulus 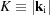 and by the unit vector 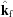 that points from the sample to the detector pixel.

Let us now designate Ψ either Ψ_i_ or Ψ_f_. Solutions of (10) separate as
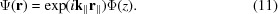
The wavevector component in the *xy* plane, 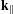, is constant. The vertical wave equation is
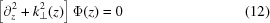
with
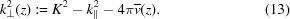
Its two solutions can be chosen to represent upwards and downwards traveling waves. So far *BornAgain*, as all extant software, only supports stepwise variations of 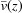.^2^ For the treatment of graded layers, see also Section 5.8.

Within layer *j*, the solution of (12) is simply
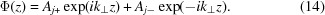
Standard continuity conditions at the layer interfaces yield linear couplings between the coefficients 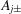. Typically, the beam comes from above, so that 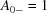 in the top (air or vacuum) layer and 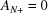 in the bottom (substrate) layer. With these normalization and boundary conditions, the coefficients are fully determined.

**Figure 6:**
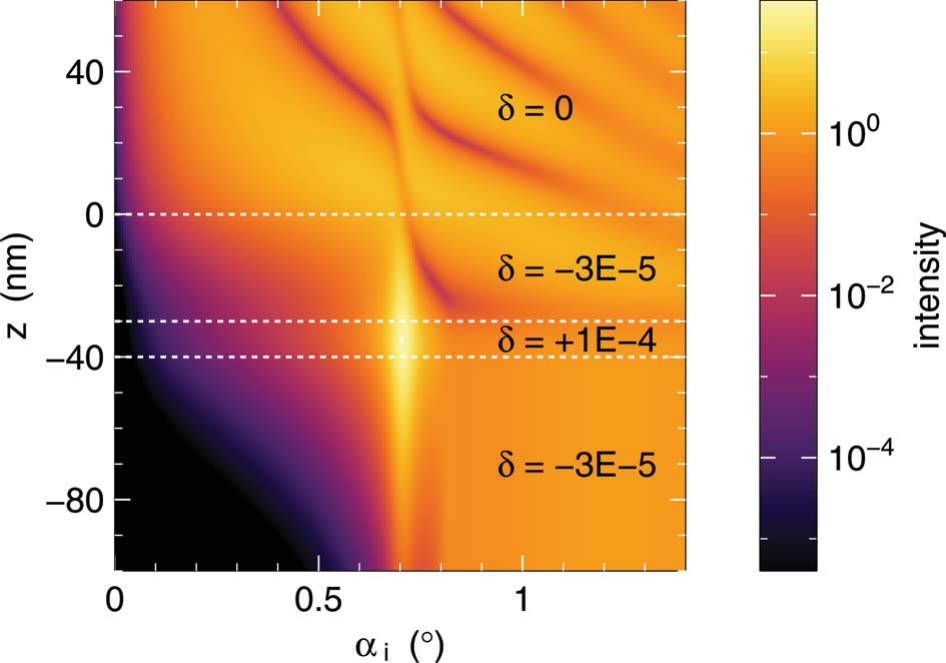
In depth-probe mode, *BornAgain* computes the refracted and transmitted beam intensity (squared amplitude) as a function of depth and incident angle. In this example, radiation with λ = 1 nm comes from vacuum (δ = 0) and enters at *z* = 0 a sample made of two layers on top of a substrate. Low-intensity bands show nodes of standing waves. An intensity maximum deep in the sample is found for α_i_ ≃ 0.7°.

The *BornAgain* Core and GUI provide an extra front end, DepthProbeSimulation, to compute the refracted and transmitted beam intensities as a function of depth and incident angle (Fig. 6 ▸). In preparing a GISAS experiment this allows users to choose the incident angle that maximizes the intensity available for scattering from within the sample.

In conclusion, the cross section (9) takes the DWBA form
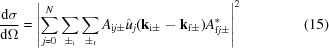
with
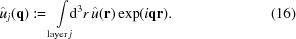
For scalar wavefunctions, all this is standard and has been implemented in several extant codes (Section 2). New in *BornAgain* is the optional simulation of polarized neutron scattering, with a 2 × 2 matrix potential 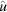 and spinor-valued coefficients *A*.

### 5.5. Materials parameterization   

Wave propagation through a multilayer (Section 5.4) is governed by the vertical wavenumber 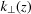 defined in (13). Re-parameterization in terms of wavenumber *K* and grazing angle α yields 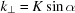 with
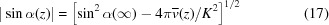


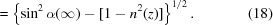
As expected, beam geometry only depends on the refractive index *n*. Since *n* is close to unity it is conveniently written as
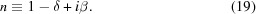
The small real numbers δ and β are the primary means to specify a material in *BornAgain* [the sign of β comes from the quantum-mechanical convention chosen in (11) and (14)].

However, *n* depends on *K*. This is of no concern at monochromatic synchrotron sources, but can have noticeable consequences at neutron and laboratory X-ray instruments. To compensate for the explicit *K* ^2^ dependence in (7), *BornAgain* supports an alternative specification of materials in terms of the SLD
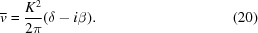
If the need arises, we will also find ways to account for the dependence of 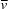 on *K*, which is most pronounced near absorption resonances.

### 5.6. Rough interfaces   

Roughness breaks the translational symmetry of an interface and thereby causes diffuse scattering into off-specular directions. As the scattered intensity is lost from the reflected or transmitted beam, reflection and transmission coefficients no longer add up to 1. The reduction of reflected and transmitted intensity is described by the Névot–Croce factor (Névot & Croce, 1980 ▸). *IsGISAXS* supports this loss factor but not the diffuse scattering.

**Figure 7:**
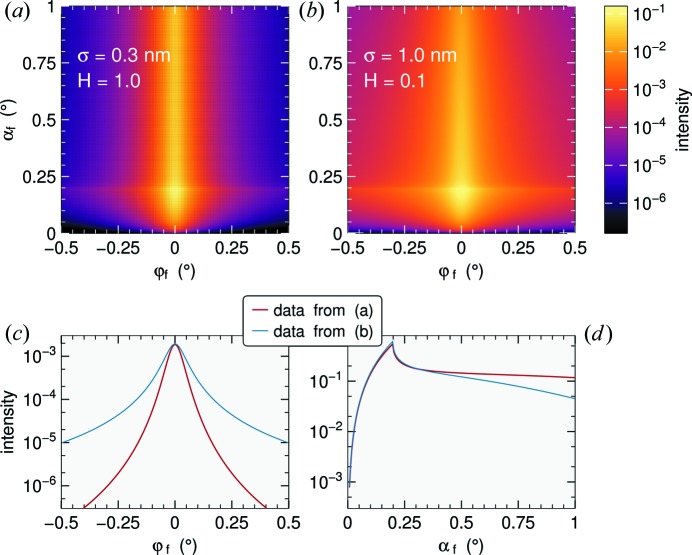
Diffuse scattering from rough interfaces. Collimated incoming beam with λ = 1 Å, α_i_ = 0.2°; substrate δ = 6 × 10^−6^, lateral correlation length ξ = 20 nm. (*a*) σ = 0.3 nm, *H* = 1; (*b*) σ = 1 nm, *H* = 0.1. In (*c*) and (*d*), the same data are integrated horizontally and vertically, respectively. The enhanced intensity below the critical angle α_c_ = 0.198°, known as the Yoneda peak, is due to scattering from the evanescent wave.

In *BornAgain*, diffuse scattering and beam attenuation are computed consistently. The roughness model is taken from the work of Schlomka *et al.* (1995 ▸). The height *h* is assumed to be a Gaussian random variable. The correlation function at in-plane distance *R* is
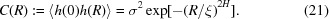
The user needs to specify the amplitude σ, the correlation length ξ and the Hurst parameter *H*. The latter is restricted to 0 < *H* ≤ 1. According to Schlomka *et al.* (1995 ▸), it defines the fractal box dimension *D* = 3 − *H* of the interface: the smaller *H* is, the more jagged is the interface. Typical simulation results are shown in Fig. 7 ▸.

If there are two or more interfaces, then their height profiles may be correlated. Following again the work of Schlomka *et al.* (1995 ▸), this is specified through a vertical cross-correlation length 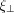 that governs the correlations between two interfaces *j* and *k*,
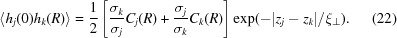



### 5.7. Particle layout (incoherent sum)   

Many GISAS studies address droplets, islands, inclusions or holes of any size from nanometre to micrometre. As pioneered by *IsGISAXS*, all such inhomogeneities are described in *BornAgain* as particles that are embedded in a material layer.

In *BornAgain*, each layer can have one or more particle layouts. The total DWBA cross section is computed as an incoherent sum over these layouts *l*, weighted by their weight *w_l_*,
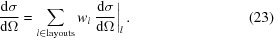
This is essentially the local monodisperse approximation of *IsGISAXS*. Typically, different layouts stand for different spatial domains of the sample. Each layout contains particles of one or several given shapes (Section 5.8). Composite particles are also possible [Fig. 3 ▸(*a*), Section 5.9]. Within one layout, interference terms arise from inter-particle correlations (Section 5.11).

### 5.8. Particle form factors (also across layers)   

**Figure 8:**
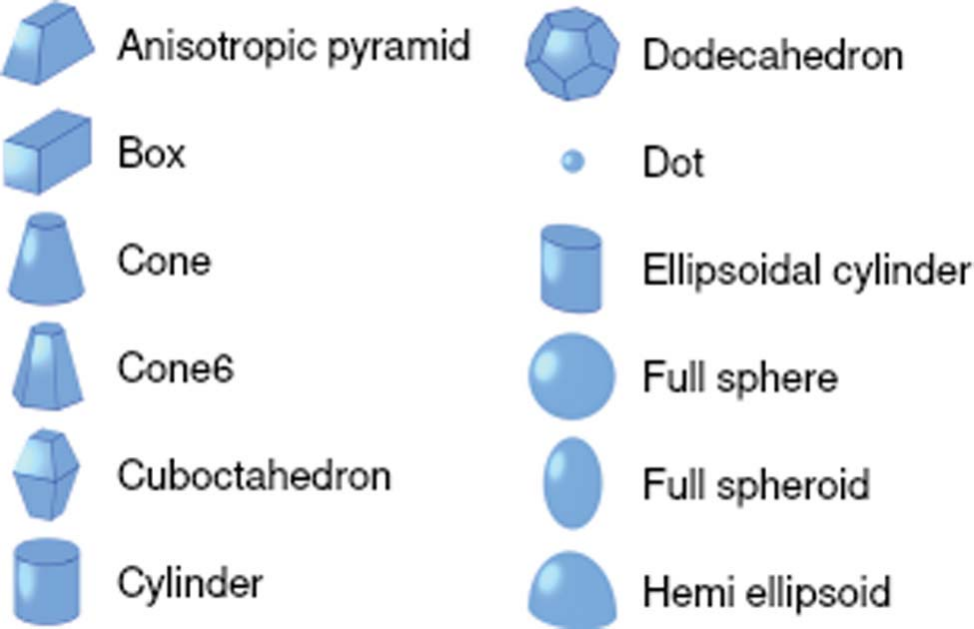
Part of the particle form-factor catalog in the GUI.

Particle shapes can be selected from a catalog of form factors. This catalog is visualized in the GUI by real-space pictures (Fig. 8 ▸); an exact description of the geometries and definitions of all parameters is given in the online documentation. *IsGISAXS* and *FitGISAXS* already supported quite a number of geometric forms. In *BornAgain*, they are all re-implemented and some more are added. The following innovations improve upon the legacy software.

**Figure 9:**
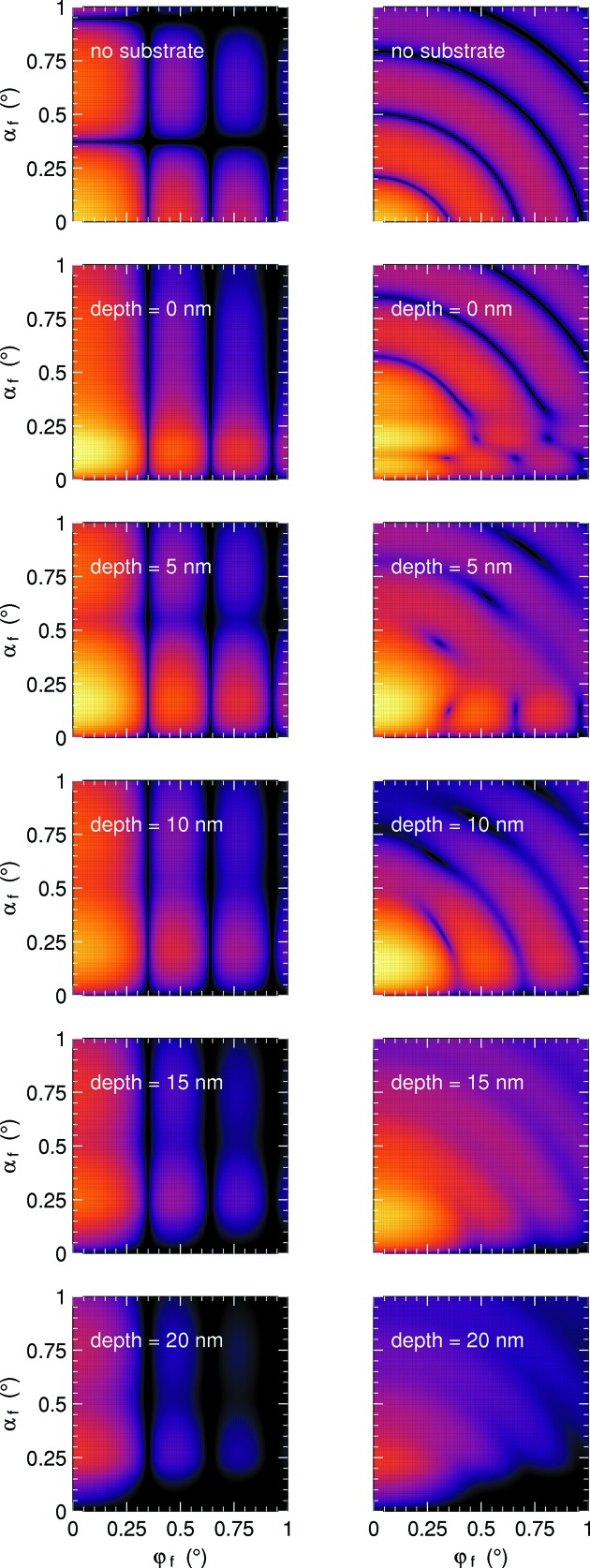
Scattering from uncorrelated cylinders (left, *R* = *H*/2 = 10 nm) and spheres (right, *R* = 10 nm); δ = 6 × 10^−4^. Collimated incoming beam with λ = 1 Å, α_i_ = 0.2°. Top row: no substrate (Born approximation). The other rows: particles are immersed to the indicated depth in an absorbing substrate with δ = 6 × 10^−6^, β = 3 × 10^−6^. The common logarithmic intensity scale covers six decades and uses the same *Matplotlib* ‘inferno’ colors as all the other color maps.

*BornAgain* fully supports absorbing media. Absorption causes a vertical damping of the incident and final wave, accounted for by an imaginary part of *q_z_*. Since particles can be freely rotated, form-factor functions admit an arbitrary complex **q** argument. Particles are allowed to cross layer interfaces, as demonstrated in Fig. 9 ▸.

Layers may consist of many sublayers to approximate a refractive index gradient. To support this, all supported particle shapes are equipped with form-factor functions for horizontal slices.^3^

Many of the supported particle shapes are polyhedra. Derivation of form factors by straightforward integration is cumbersome and yields expressions that contain removable singularities, causing numeric instabilities. We therefore preferred a generic solution (Wuttke, 2017 ▸), based on the Gauss and Stokes integral theorems, and parameterized in terms of edge topology and vertex coordinates. Near the singularities series expansions are used to avoid cancelation. To demonstrate the power of this method, we implemented the form factors of the regular dodecahedron and icosahedron. Other polyhedra can be supported as the need arises.

For large particles, keep in mind Section 5.3 on the evaluation of narrow peaks in a finite detector grid.

### 5.9. Particle composition   

A particle composition combines simple particles (with hard-coded form factors as described above) into a more complicated particle. This can be used to create arbitrary geometric shapes, to account for different SLDs (*e.g.* hard core and soft shell) and to construct a basis for a crystalline unit cell.

The resulting composed particle allows the same operations as simple particles: it can be further composed, rotated, translated and added to a layer’s particle layout. The scattering from a composed particle will correctly account for the coherent interference between its constituents.

### 5.10. Parameter distribution   

**Figure 10:**
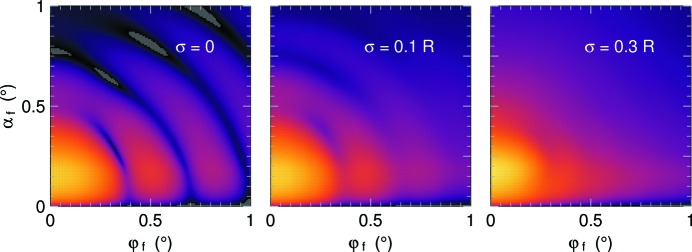
Scattering from immersed spheres as in Fig. 9 ▸, at depth 10 nm. The spheres now have a Gaussian size distribution, with standard deviations σ from 0 to 0.3*R*. Logarithmic intensity scale, covering six decades, as before.

In many applications, the size and form parameters of nanoparticles have no uniform fixed values but follow some random distribution. GISAS patterns of such polydisperse systems are smoother than in the monodisperse limiting case; in particular, sharp minima are averaged out. This is exemplified in Fig. 10 ▸.

In *BornAgain*, stochastic distributions can be assigned to any particle geometry parameter, and to many other sample or instrument parameters, like interface roughness or incident wavelength. Available distributions include rectangular, trapezoid, Gaussian, Lorentzian and log-normal; for those with infinite support, lower and upper cutoffs can be set. To make one parameter proportional to another, one can impose a link.

If any parameter has a stochastic distribution, then the average DWBA cross section is computed from a configurable number of weighted equidistant parameter samples. In the concrete computation, however, a difference is made between particle geometry parameters and all other parameters. Sampled particles are all inserted in one and the same particle layout (Section 5.7) so that the DWBA cross section is obtained as a coherent sum. For all other sample and instrument parameters, the GISAS pattern is computed as an incoherent average over the parameter samples. This generalizes the parameter handling of *IsGISAXS* (Section 2.2.4 in Lazzari, 2006 ▸).

### 5.11. Particle correlations (interference functions)   

Particles, in the wide sense adopted here (Section 5.7), can aggregate in quite different structures, from complete disorder to crystalline lattices. Following *IsGISAXS* terminology, these models are implemented under the name interference function.

If no inter-particle correlation is specified, then *BornAgain* assumes perfect disorder, with structure factor 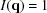, which of course is physical only for dilute systems. The dilute limit is safely reached when inter-particle distances are larger than 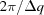, where Δ*q* is the resolution width.

If excluded-volume interactions matter, then the next simplest model approximates particles as hard spheres, or hard discs in two dimensions. As GISAS is a surface technique, it is unsurprising that user demand so far has only been articulated for the 2D hard-disc model. Analytical solutions of the Percus–Yevick equation, however, seem to exist only for certain odd dimensions. Therefore we implemented the analytical approximation of Ripoll & Tejero (1995 ▸), which is in good accord with numeric results, though somewhat less so at packaging fractions above 0.5.

The next degree of ordering is represented by paracrystals (Hosemann, 1951 ▸). We implemented the radial and the 2D paracrystal exactly as in *IsGISAXS* (Lazzari, 2002 ▸, 2006 ▸). For the radial paracrystal, one can choose between the decoupling and the size-spacing correlation approximations.

### 5.12. Crystals, mesocrystals, superlattices   

**Figure 11:**
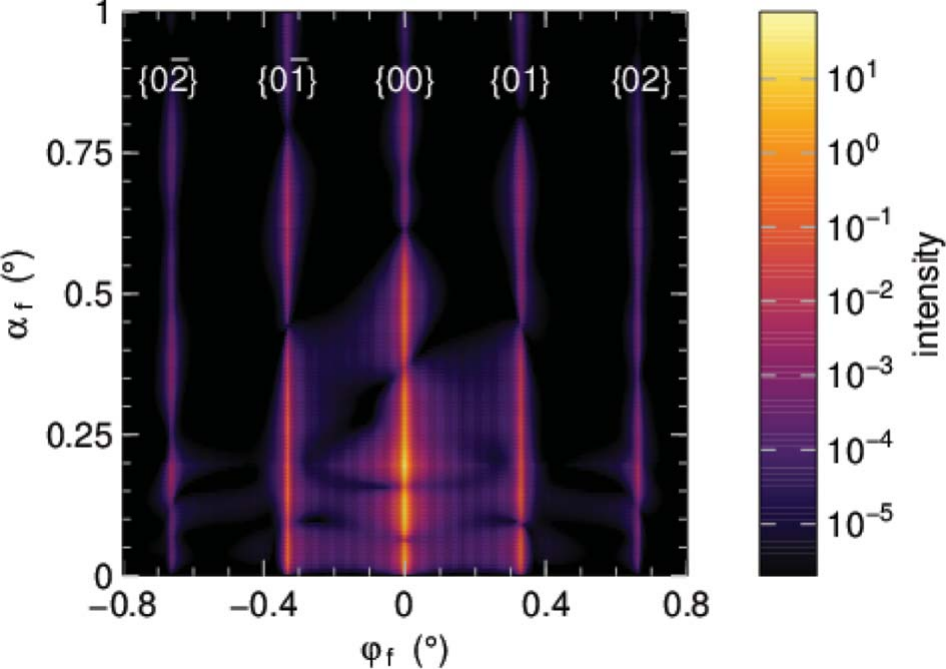
GISAS pattern of the sample model introduced above in Figs. 2 ▸ and 3 ▸: spherical nanoparticles (*R* = 10 nm, δ = 6 × 10^−4^), forming a hexagonal dense bilayer on top of a substrate (δ = 6 × 10^−6^), for a highly symmetric lattice orientation ({11} along *x*). The incident beam has λ = 1 Å, α_i_ = 0.2°. Bragg rods have been indexed manually. The finite horizontal extension of the rods is imposed through a decay function with correlation length 400 nm. The slight breaking of the mirror symmetry in φ_f_ is an artifact of our sample model with its rigid two-particle motif.

Crystalline lattice structures in one, two and three dimensions can be specified in a straightforward way by supplying the one, two or three primitive vectors of the primitive Bravais lattice.^4^ Non-primitive bases are to be specified in the form of particle composition motifs. Fig. 11 ▸ shows the simulated GISAS pattern for the hexagonal bilayer introduced above in Figs. 2 ▸ and 3 ▸. Note that Bragg rods other than {00} are only observed for certain lattice orientations.

As anticipated in Section 5.3, the delta-shaped diffraction peaks of a perfect crystal are incompatible with our straightforward way of sampling the GISAS cross section at discrete **q**. Following *IsGISAXS* [Section 2.5.2 of Lazzari (2006 ▸)], *BornAgain* provides two ways to widen crystalline diffraction peaks: (i) the exact computation of finite size effects is most convincing for the modeling of well-known lithographic structures; (ii) in most other cases, finite-size effects are dominated by crystal defects and/or by the finite coherence length of the scattering apparatus. To model these imperfections, the delta functions are overridden *ad hoc* by finite peak shapes, called FTDecayFunctions because they can be construed, at least in a good approximation, as a Fourier transform of some decay of correlations in real space.

Mesocrystals are small crystalline aggregates of nanoparticles, typically formed by colloidal crystallization (*e.g.* Cölfen & Antonietti, 2005 ▸). If the size and distance of the mesocrystals are of a suitable scale, then GISAS is able to provide information on the size and outer shape of mesocrystals, and about their correlations. *BornAgain* supports mesocrystals in huge generality: to specify mesocrystal shape and inter-mesocrystal correlations, one can use the form factors and interference functions of Sections 5.8–5.11. For instance, one could model a hard-disc arrangement of cylindrical mesocrystals that consist of a face-centered cubic lattice of spherical nanoparticles. (Diffuse scattering from mesocrystals is not yet simulated, but this is expected to be implemented in a forthcoming release.)

To describe certain man-made surface structures, *Born­Again* also supports superlattices, which are 2D lattices of finite lattices.

Periodic gratings can be modeled as a 1D lattice of ripples or other elongated particles like pyramids or boxes. Of course only single scattering in the DWBA is simulated. Completely different computations are needed to account for higher-order dynamic scattering effects (Ashkar *et al.*, 2010 ▸; Soltwisch *et al.*, 2017 ▸).

### 5.13. Magnetism and neutron polarization   

The DWBA implementation of *BornAgain* fully supports polarized neutron propagation. With the exception of rough interfaces, all scattering models in *BornAgain* can be simulated for polarized neutrons. Specifically, this means that magnetic layers and magnetic particles can be simulated with a polarized neutron beam and optional polarization analysis at the detector. The underlying mechanism is essentially that of Kentzinger *et al.* (2008 ▸) and Toperverg (2015 ▸, and references 9, 10, 21, 45 therein). Other magnetic sample models will be implemented as requested by users. In particular, we expect interest in modeling domains in magnetic layers or magnetic roughness at layer interfaces.

By default, *BornAgain* assumes that the incoming beam is unpolarized and that there is no polarization analyzer after the sample. This is all one needs for X-rays and for nuclear scattering of neutrons. If there is magnetic neutron scattering, then *BornAgain* can be used to simulate all the functionality of a polarizing reflectometer, like MARIA at MLZ Garching (Mattauch *et al.*, 2018 ▸). The incident polarization is to be specified as a Bloch vector, which allows one to encode any density matrix, but is more intuitive in that it allows one to directly read off the polarization direction and the extent of mixing. The polarization analyzer is specified in terms of orientation, efficiency and transmission.

**Figure 12:**
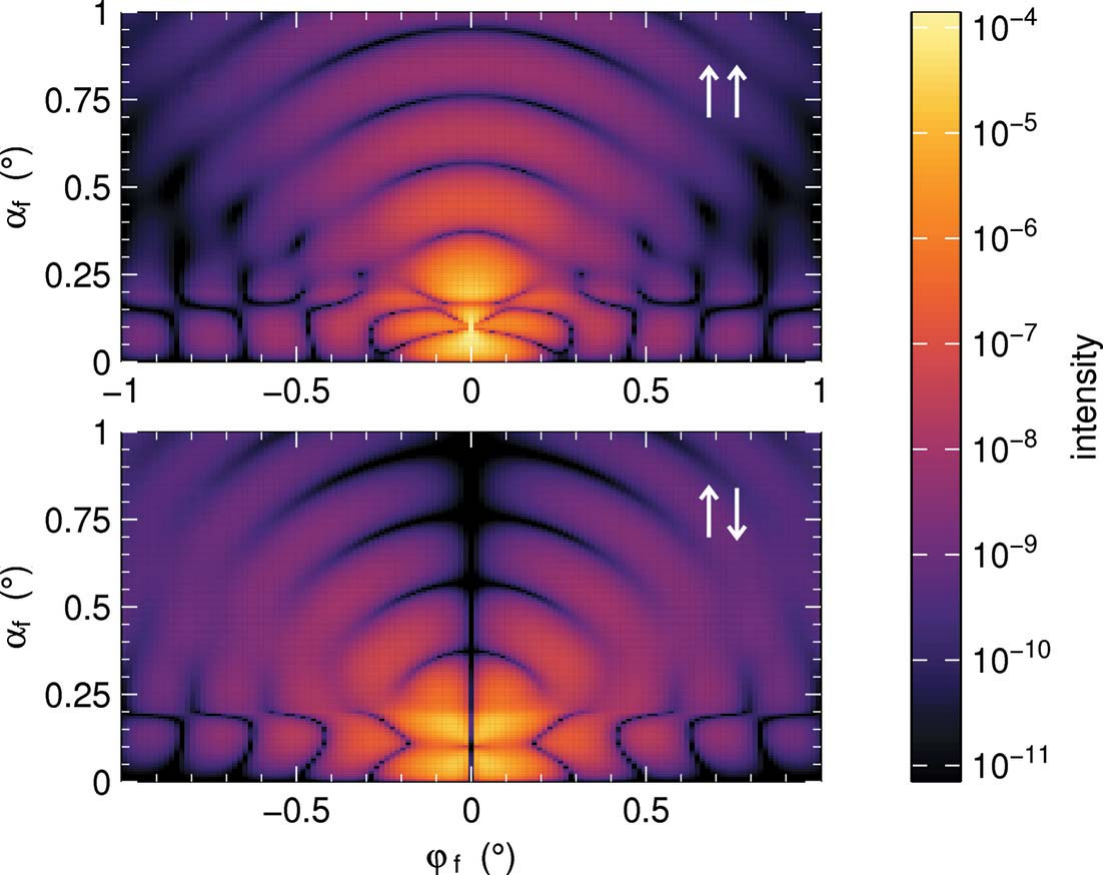
Polarized GISANS from vertically magnetized spherical nanoparticles (*R* = 30 nm, δ = 6 × 10^−7^, *M* = 400 kA m^−1^) on top of a substrate (δ = 6 × 10^−6^). The incident beam has λ = 2 Å, α_i_ = 0.1°. The two simulations correspond to scattering (*a*) without and (*b*) with spin flip, assuming perfect vertical beam polarization and perfect polarization analysis.

An example of scattering by magnetized nanospheres is given in Fig. 12 ▸. The scattering cross section comprises nuclear, magnetic and interference terms. When polarizers are set for detection of spin-flip scattering, then nuclear scattering is suppressed; one only sees scattering from the magnetization component 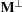 perpendicular to **q** (*e.g.* Chatterji, 2006 ▸). This explains the gap around φ_f_ = 0 in Fig. 12 ▸(*b*).

## 6. Data processing and fitting   

### 6.1. Data import, visualization, masking and slicing   

*BornAgain* can read detector images from tiff files, from plain ASCII tables or from an internal ASCII storage format. For all other input, users are referred to the Python library *FabIO* (Knudsen *et al.*, 2013 ▸) that can convert detector images from a huge variety of data formats used at X-ray instruments.

When the intensities supplied on input are all integers, and no error estimate is given, then it is presumed that the intensity *D_i_* represents the number of independently scattered particles counted in pixel *i*, and the error estimate δ*D_i_* is taken from the Poisson standard deviation (*D_i_*)^1/2^.

Experimental scattering intensity histograms are plotted in the same way as simulated ones, *i.e.* as 2D color maps. In fitting mode, the GUI shows experimental and simulated data side by side, complemented by a difference image and a fit progress plot. Plots can be exported from the GUI as PNG graphics files. Under Python, the example scripts show how to plot data and simulations. From the *Matplotlib* pop-up window, plots can be exported to a variety of graphics formats, including PNG, TIFF and PDF.

To prepare data for 2D fitting it can be necessary to mask parts of the detector area, for instance those shadowed by the beamstop. In the GUI this can be done by drawing ellipses or polygonal contours. Data can also be projected into 1D histograms. This may be desired for conventional function plotting [as in Figs. 7 ▸(*c*) and 7 ▸(*d*)], and for the direct, sensitive comparison of data and fits.

### 6.2. Fit engines   

As with other *BornAgain* functionality, the fitting of parametric models to experimental data can also be steered from either the Python API or, to some extent, the GUI. To get started, one ties a model to a data set and chooses a residual function *R_i_*, which can be the plain difference between data *D_i_* and parametric model *f_i_*(**P**), or the error-weighted difference 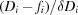, or the logarithm thereof. The default objective function is then the sum of squared residuals, 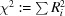.

At the core of the fitting procedure a minimizer algorithm searches for a minimum of χ^2^ as a function of the parameter vector **P**. *BornAgain* comes with a choice of local and global minimizers, taken from several libraries, as specified in Section 3.5.

We wrote a wrapper that allows all minimizers to be called in the same way from the GUI or from Python scripts. The API of this wrapper closely follows that of the Python libraries *bumps* and *lmfit*. This allows users of the *BornAgain* Python API to choose either one of those Python minimizers, or one of the minimizers included in *BornAgain*, or even to concatenate different minimizers. Some of the minimizers allow or even require that parameters be restricted to finite intervals. Additional constraints, like coupling between parameters, can be imposed from the Python API.

### 6.3. Limitations of automatic fitting   

Automatic fitting of parametric models to experimental data is more difficult for GISAS than for most other scattering methods, and often is outright impossible. Many published GISAS analyses are only half-quantitative: they show qualitative agreement between the model and data, and extract some parameter values, but do not claim a ‘best fit’. We see two fundamental reasons for this: the high number of model parameters and the prevalence of systematic over stochastic uncertainties.

The high number of model parameters comes from the complexity of typical 2D samples. It is exacerbated when statistical distributions are needed to cope with fluctuations and imperfections caused by the intrinsic difficulties of thin-film preparation and alignment.

Typical GISAS patterns vary over decades in intensity, yet are much weaker than the reflected or transmitted direct beam. In such a situation, any inaccuracy in accounting for the strong signal components (*e.g.* the halos of the direct beam) will result in huge systematic uncertainty regarding the weak components. This then invalidates the objective function of a fit algorithm that depends on stochastic uncertainty estimates.

Since these difficulties overburden available automatisms, human users have to take back control. Still there is an important role for software, namely to provide versatile support for heuristic adjustments. *BornAgain* does this in particular through its masking and slicing facilities (Section 6.1).

## 7. Published usage   

As of July 2019, *BornAgain* has been used for data analysis in 19 published GISAXS and GISANS studies (not counting reviews, method papers and other publications that just mention *BornAgain* in passing). Four of these papers have been written in cooperation with one of us, some more acknowledge our help with *BornAgain*, some authors have attended a *BornAgain* school, but more than half of the published papers appeared without any personal involvement of ours: the authors just downloaded *BornAgain* and found their way to use it.

The studied samples represent the enormous breadth of modern surface science. They involve films of water (Gutfreund *et al.*, 2016 ▸), alkane (Fontaine *et al.*, 2018 ▸), lipid (Nylander *et al.*, 2017 ▸), organic semiconductor (Zykov *et al.*, 2017 ▸) and inorganic semiconductor (Highland *et al.*, 2017 ▸; Singh *et al.*, 2017 ▸); microgels (Kyrey *et al.*, 2018 ▸) and microemulsions (Frielinghaus *et al.*, 2017 ▸); templated (Li-Destri *et al.*, 2016 ▸) and self-assembled polymer structures (Berezkin *et al.*, 2018 ▸; Glavic *et al.*, 2018 ▸; Xie *et al.*, 2018 ▸); nanocolumns growing from vapor deposition (Haddad *et al.*, 2016 ▸); Au nanoparticles during CO oxidation (Odarchenko *et al.*, 2018 ▸); C_60_ monolayer islands (Kowarik, 2017 ▸); magnetic nanoparticles (Ukleev *et al.*, 2016 ▸, 2017 ▸) and magnetic films (Merkel *et al.*, 2015 ▸; Glavic *et al.*, 2018 ▸); lithographic gratings (Pflüger *et al.*, 2019 ▸); and sputtered multilayers (Frielinghaus *et al.*, 2017 ▸).

*BornAgain *is used in very different ways in these papers. Experiment and simulation are compared in the form of 2D detector images or of 1D projections. In most studies, the *BornAgain* model is manually tuned to reach good qualitative agreement with the experiment. Automatic fits are mainly done for 1D slices. Typically, the simulation yields sharper peaks than the experiment (Ukleev *et al.*, 2016 ▸, 2017 ▸; Nylander *et al.*, 2017 ▸). It remains to be seen whether this can be overcome by realistic modeling of instrument and/or sample imperfections. Pflüger *et al.* (2019 ▸) make the opposite choice: they do not even attempt to simulate a strong and nontrivial diffuse scattering; they fully concentrate on the sharp diffraction rings – which makes the accord of experiment and simulation no less impressive.

To demonstrate that a qualitative agreement between simulation and experiment is meaningful and informative, it can be helpful to present simulated detector images for modified models (Fontaine *et al.*, 2018 ▸). Also, it can be very instructive to follow experiment and simulation through some parameter variation (Li-Destri *et al.*, 2016 ▸; Singh *et al.*, 2017 ▸; Berezkin *et al.*, 2018 ▸).

## 8. Outlook   

To conclude, we briefly look beyond the current capabilities of *BornAgain*. Right now we are extending *BornAgain* towards reflectometry and off-specular scattering (Section 8.1). Application to ordinary small-angle scattering is possible though not actively promoted (Section 8.2). Finally, we mention alternatives to multi-parameter model fitting (Section 8.3).

### 8.1. Reflectometry and off-specular scattering   

As mentioned in Section 1, we are currently working on extending *BornAgain* to specular reflectometry and off-specular scattering, and will report in more detail on this in a sequel publication. Specular reflectometry is all about refracted and reflected intensities in a multilayer. The very same computations are needed by the DWBA (Section 5.4). Therefore, the sample model, the data structures and the core algorithms of *BornAgain* have long been ready for reflectometry simulations. Extra development effort is only needed to expose this functionality through convenient graphical and Python user interfaces. Similarly, off-specular scattering requires little more than an appropriate representation of yet another scan mode.

For the specular case, *BornAgain* will compete with quite a number of other data analysis programs: *Aurore* (Gerelli, 2016 ▸), *GenX* (Björck & Andersson, 2007 ▸), *Motofit* (Nelson, 2006 ▸, 2010 ▸), *refnx* (Nelson & Prescott, 2019 ▸), *RasCAL* (Hughes, 2014 ▸) and *Refl1D* (Kienzle *et al.*, 2018 ▸). Against these, *BornAgain* stands out by its full support for neutron polarization, by its capability to derive scattering-length gradings from a rich and versatile particle decoration model, and not least by being institutionally supported. For users and instrument scientists of modern reflectometers, it will be convenient to have one and the same software cover all three scan modes. Ideally, this offer will encourage specular-only users to also explore the off-specular information, which a multi-detector captures anyway.

### 8.2. Small-angle scattering   

*BornAgain*, as with any GISAS code, can easily be adapted to fit and simulate ordinary small-angle scattering (SAS) by rotating the incoming beam and detector location. Since there will be almost no reflection from interfaces, the coefficients 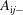 and 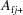 in (14) can be set to zero, so that no sums over 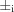 and 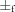 must be computed in (15). This of course corresponds to replacing the DWBA by the ordinary Born approximation – an option which *BornAgain* anyhow offers for testing and teaching purposes.

However, there exist other institutionally supported codes for SAS analysis. In particular, within the European collaboration SINE2020 (see Section 1 and Acknowledgments) it was agreed to concentrate efforts on the software *SasView* (Doucet *et al.*, 2018 ▸). Therefore, we do not promote *Born­Again* as a SAS software, and we do not offer user support for this application domain, except where *BornAgain* offers pertinent functionality that is lacking in *SasView*. This is especially the case for magnetic scattering, and for scattering from ordered nano- and mesoscale materials (Förster *et al.*, 2011 ▸); to support research in this latter field, we extended our lattice models to three dimensions, replicating functionality of the soft-matter SAS software *Scatter* (Förster *et al.*, 2010 ▸).

### 8.3. Beyond fitting   

As discussed in Section 6.3, model adjustment by automatic fitting becomes difficult or outright impossible if there are too many free parameters or if strong model components overshadow weak components and systematic uncertainties outweigh stochastic ones. One possible approach to this problem consists of reducing the task of data analysis by advanced data reduction, *e.g.* by ‘unwarping’ the four terms of the GISAS cross section (Liu & Yager, 2018 ▸). This may be worth a re-implementation within *BornAgain*. In reflectometry, the parameter-free determination of depth profiles by inversion of experimental curves has already been explored a number of times (de Haan *et al.*, 1996 ▸; Majkrzak *et al.*, 1997 ▸; Bushuev *et al.*, 2002 ▸; Sutyrin & Prokhorov, 2006 ▸).

Another approach to cope with underdetermined multi-parameter models is machine learning. First applications to GISAS are promising (Wang *et al.*, 2017 ▸; Liu *et al.*, 2019 ▸). Well-trained deep neural networks can select models and deliver model parameters in microseconds, whereas manual fitting takes days at best. The precondition for this is a rich set of labeled training data. These data can be generated by running *BornAgain* on a powerful CPU cluster for a huge variety of models. We are currently exploring this idea in a pilot study (Van Herck *et al.*, in preparation).

## Footnotes

1 If the intensity is peaked at φ_f_ = 0° (like in Fig. 11), then it is particularly helpful to compare the GISAS pattern on a symmetric φ_f_ axis with *N* versus *N* + 1 bins. In the one case, for an odd number of bins, one bin is centered at φ_f_ = 0°, and therefore will overestimate the average intensity; in the opposite case, the distance from 0° to the nearest bin center is maximum, and therefore the average intensity will be maximally underestimated.

2 Analytical solutions are also known for a linear dependence of  on *z* (Vallee & Soares, 2010 ▸), but have not yet been considered for implementation.

3 At present, there is one restriction though: some particles cannot be sliced and tilted at the same time. Should this functionality be required for some realistic sample model, we will implement it using either numeric integration of 2D form factors or numeric convolution of 3D form factors (Venkatakrishnan *et al.*, 2016 ▸).

4 They are implemented in the classes InterferenceFunction1DLattice *etc*., whereas the class Crystal is only meant to be used within a MesoCrystal.
